# Time-dependent changes in P2Y12 reaction unit values for predicting the different types of cardiovascular events in patients with ischemic heart disease

**DOI:** 10.1007/s00380-023-02279-0

**Published:** 2023-06-15

**Authors:** Takatoku Aizawa, Yasunori Inoue, Satoshi Ito, Satoshi Morimoto, Kazuo Ogawa, Tomohisa Nagoshi, Kosuke Minai, Takayuki Ogawa, Makoto Kawai, Michihiro Yoshimura

**Affiliations:** Division of Cardiology, Department of Internal Medicine, The Jikei University School of Medicine, 3-25-8 Nishi-shinbashi, Minato-ku, Tokyo, 105-8461 Japan

**Keywords:** P2Y12 reaction unit, Time-dependent receiver-operating characteristic analysis, Optimal cut-off value, Major adverse cardiovascular event

## Abstract

Several studies have investigated the association between P2Y12 reaction unit (PRU) value and major adverse cardiovascular events (MACEs) in patients with ischemic heart disease, but there is no well-established consensus on the utility of PRU value. Furthermore, the optimal PRU cut-off value varied with studies. One reason may be that the endpoints and observation periods differed, depending on the study. This study aimed to investigate the optimal cut-off and predictive ability of the PRU value for predicting cardiovascular events, while considering different endpoints and observation periods. We surveyed a total of 338 patients receiving P2Y12 inhibitors and measured PRU during cardiac catheterization. Using time-dependent receiver operating characteristic analysis, we evaluated the cut-off and area under curve (AUC) of the PRU value for two MACEs (MACE ①: composite of death, myocardial infarction, stent thrombosis, and cerebral infarction; MACE ②: composite of MACE ① and target vessel revascularization) at 6, 12, 24 and 36 months after cardiac catheterization. MACE ① occurred in 18 cases and MACE ② in 32 cases. The PRU cut-off values at 6, 12, 24, and 36 months were 257, 238, 217, and 216, respectively, for MACE ① and 250, 238, 209, and 204, respectively, for MACE ②. The AUCs at 6, 12, 24, and 36 months were 0.753, 0.832, 0.718, and 0.717, respectively, for MACE ① and 0.724, 0.722, 0.664, and 0.682, respectively, for MACE ②. The optimal cut-off and predictive ability of PRU values for cardiovascular events varied depending on different endpoints and duration of the observation periods. A relatively high PRU value is effective for short-term event suppression, but a low value is required for long-term event suppression.

## Introduction

In patients with ischemic heart disease, high treatment platelet reactivity (HPR) has been found to be significantly associated with cardiovascular events on P2Y12 inhibitor therapy [1, 2]. The VerifyNow P2Y12 assay is a reliable, fast, and sensitive test suitable for monitoring platelet inhibition during P2Y12 inhibitor therapy [3–5]. Several studies have investigated the association between the P2Y12 reaction unit (PRU) value, as measured by the VerifyNow P2Y12 assay, and cardiovascular events in patients with ischemic heart disease. However, there is no well-established consensus regarding the availability and reliability of PRU values. Previous reports have demonstrated the association between the PRU value and cardiovascular events [6–13]. However, some reports have refuted the association between the PRU value and cardiovascular events [14–17]. Therefore, there are conflicting reports regarding the association between PRU value and cardiovascular events. One potential reason for this might be the difference in the endpoints and observation periods in numerous studies. Few investigations have simultaneously examined the association between the PRU cut-off values for cardiovascular events and different endpoints/observation periods.

Receiver operating characteristic (ROC) analysis is a commonly used method for evaluating the cut-off value for continuous variables for a single endpoint event. However, a typical ROC analysis cannot integrate the effect of time in the analysis. Previous studies have used ordinary ROC analyses to investigate the PRU cut-off value. However, the observation duration could significantly impact the study results and affect the occurrence of cardiovascular events. If the analysis is performed after considering the course of time, it would be necessary to take the drop-out cases into account as well. Time-dependent ROC analysis enables estimation of the cut-off values of independent variables for the occurrence of endpoints, while considering drop-out cases over the course of time [18]. The predictive ability of the cut-off value is evaluated using the area under the curve (AUC). Therefore, it is meaningful to evaluate the predictive ability of independent variables for event outcomes incorporating the course of time, using time-dependent ROC analysis.

This study aimed to investigate the optimal cut-off and predictive ability of the PRU value for predicting cardiovascular events, considering both the differences in endpoints and observation periods using time-dependent ROC analysis.

## Materials and methods

### Study population

The study population comprised 338 patients with ischemic heart disease receiving clopidogrel (75 mg/day) or prasugrel (3.75 mg/day) who underwent cardiac catheterization at The Jikei University Hospital from September 2017 to January 2020. The exclusion criteria were P2Y12 inhibitor taken for less than 7 days or a hematocrit value ˂ 20% or ˃ 60%.

This study was conducted in accordance with the principles expressed in the Declaration of Helsinki and was approved by the Medical Ethics Committee of Jikei University School of Medicine [24-355[7121]. This was a retrospective study, and informed consent was not obtained from each patient. Instead, according to our institution’s routine ethical regulations, we posted a notice about the study design and contact information at a public location in our institution. In this public notification, we ensured that patients had the opportunity to refuse participation (opt-out) in this study.

### Clopidogrel or prasugrel administration

All participants were being managed on a P2Y12 inhibitor because of a history of PCI or because they were scheduled for PCI. The duration of P2Y12 inhibitor administration after cardiac catheterization was at the discretion of the treating physician in accordance with the Japanese guidelines. In addition, there were no stipulations regarding the choice of P2Y12 inhibitor (clopidogrel or prasugrel), which was, therefore, chosen at the discretion of the physician.

### Blood sampling and measurement of platelet function

We collected blood samples from the arterial sheath during cardiac catheterization, which were immediately transferred into blood tubes. The effect of the P2Y12 inhibitor was assessed using the VerifyNow P2Y12 assay (Accumetrics, San Diego, CA, USA) between 10 min and 4 h after equilibration. The VerifyNow P2Y12 assay is a point-of-care test designed to directly measure the effects of drugs on the P2Y12 receptor [4]. This device is a turbidimetric optical detection system that measures light transmittance. P2Y12 inhibitor responsiveness was assessed by ADP-induced platelet aggregation and recorded as PRUs. Routine biochemical analyses, such as those for electrolytes, renal function, and liver function, as well as lipid and glucose profiles, were performed in a central laboratory at our hospital.

### Endpoints

The study endpoints were first major adverse cardiovascular events (MACEs), and we defined two MACEs, namely, MACE ① and MACE ②. MACE ① was defined as the composite of all-cause death, non-fatal myocardial infarction, stent thrombosis, and non-fatal cerebral infarction, and MACE ② was defined as the composite of all-cause death, non-fatal myocardial infarction, stent thrombosis, non-fatal cerebral infarction, and target vessel revascularization (TVR). We evaluated the occurrence of endpoints at 6, 12, 24, and 36 months after cardiac catheterization. Non-fatal myocardial infarction was defined as an increase in myocardial deviant enzymes (creatine kinase MB, troponin I, or troponin T) and at least one of the following: symptoms of myocardial ischemia, novel ischemic electrocardiographic changes, evidence of novel local myocardial wall motion abnormalities consistent with myocardial ischemia on imaging, and evidence of intracoronary thrombus on coronary angiography [19]. TVR was defined as performing revascularization by PCI or coronary artery bypass grafting (CABG) of the target vessel.

In addition, we investigated the first major bleeding event. Bleeding was defined according to the Bleeding Academic Research Consortium (BARC) criteria [20]. Major bleeding was defined as BARC type 3 or 5.

### Statistical analysis

Continuous variables were expressed as mean ± standard deviation (SD) values or median values with the associated range. Categorical variables were expressed as percentages. The occurrence of events after cardiac catheterization was represented by the Kaplan–Meier curve. The survival analysis between the two groups up to the first event at 6, 12, 24, and 36 months was performed using Cox regression models. *P* < 0.05 was considered statistically significant. All statistical analyses were performed using SPSS Statistics version 25.0 (SPSS Inc., Chicago, IL, USA).

Time-dependent ROC analysis was used in this study. Ordinary ROC analysis is a well-established statistical method to evaluate the strength of the correlation between the independent variable, which is a continuous variable, and the outcome, which is a dichotomous variable. However, the ROC curve assumes that the disease outcome does not change over time. Nonetheless, it is important to consider that disease outcomes could be time dependent. Therefore, time-dependent ROC curve analysis was used to evaluate the predictive ability of the independent variable for time-dependent disease outcomes [18]. In time-dependent ROC curve analysis, the individual disease outcomes are observed and updated at each time point. This curve is created using the sensitivity (t) and 1-specificity (t) obtained from the various cut-off values of the independent variables at time (t). A time-dependent ROC curve can be drawn at any time (t) using these measured values. The Youden index method is used to calculate the optimal cut-off value. The predictive ability of the independent variables can be accurately evaluated by constructing ROC curves at several time points. Therefore, time-dependent ROC curve analysis is an efficient statistical method for precise evaluation of the outcome of independent variables. The time-dependent ROC was performed using EZR (Saitama Medical Center, Jichi Medical University, Saitama, Japan), which is a graphical user interface for R (The R Foundation for Statistical Computing, Vienna, Austria) [21]. More precisely, it is a modified version of R commander designed to add statistical functions frequently used in biostatistics. We used the “survival ROC” package, written for R, to assess the optimal PRU cut-off value for preventing cardiovascular events [18].

## Results

### Clinical characteristics and lesion characteristics

Among 338 study participants, 314 (92.9%) patients had stable angina and 24 (7.1%) had acute coronary syndrome. The baseline characteristics of the overall study population are presented in Table 1. There were 181 patients taking clopidogrel (53.6%) and 157 patients taking prasugrel (46.4%). In addition, most of the patients were being managed using aspirin (94.4%), statins (89.9%), and proton pump inhibitors (89.6%). Mean duration of treatment with P2Y12 inhibitor after cardiac catheterization was 305 days. The percentage of P2Y12 inhibitor use at 6 months, 12 months, 24 months, and 36 months after cardiac catheterization was 66.6%, 35.3%, 8.7%, 2.0%, respectively.

**Table 1:** Clinical characteristics

Characteristic	Overall (n = 338)
	Number (%) or mean ± SD or median [interquartile range]
Sex, male	300 (88.8)
Age (years)	65.5 ± 11.0
BMI (kg/m^2^)	24.7 ± 3.6
Current smoking	60 (17.8)
Family history of IHD	104 (30.8)
Hb (g/dL)	13.6 ± 1.8
Platelet count (× 10^3^/μL)	217.5 ± 67.5
Creatinine (mg/dL)	1.15 ± 1.52
eGFR (mL/min/1.73 m^2^)	69.6 ± 22.7
UA (mg/dL)	5.7 ± 1.4
FBS (mg/dL)	112.9 ± 32.5
HbA1c (%)	6.3 ± 1.0
TG (mg/dL)	117.8 ± 64.1
HDL-C (mg/dL)	50.0 ± 14.2
LDL-C (mg/dL)	86.4 ± 23.4
CRP (mg/dL)	0.07 [0.03–0.19]
BNP (pg/mL)	31.3 [15.2–106.7]
PRU	177.2 ± 68.6
Old myocardial infarction	142 (36.9)
Cardiomyopathy	15 (4.4)
Arrhythmia	35 (10.4)
Valvular heart disease	12 (3.6)
Hypertension	252 (74.6)
Diabetes mellitus	130 (38.5)
Dyslipidemia	259 (76.6)
Hemodialysis	14 (4.1)
*Medication*	
Clopidogrel	181 (53.6)
Prasugrel	157 (46.4)
Aspirin	319 (94.4)
Anticoagulant agent	24 (7.1)
ACE inhibitors	123 (36.4)
ARBs	137 (40.5)
Beta blockers	209 (61.8)
Calcium channel blockers	198 (58.6)
Diuretics	69 (20.4)
Statins	304 (89.9)
PPI	303 (89.6)
Oral antidiabetic agents	75 (22.2)
Insulin	26 (7.7)

SD, standard deviation; BMI, body mass index; IHD, ischemic heart disease; Hb, hemoglobin; eGFR, estimated glomerular filtration rate; UA, uric acid; FBS, fasting blood sugar; HbA1c, hemoglobin A1c; TG, triglyceride; HDL-C, high-density lipoprotein cholesterol; LDL-C, low-density lipoprotein cholesterol; CRP, C-reactive protein; BNP, B-type natriuretic peptide; PRU, P2Y12 reaction unit; ACE, angiotensin-converting enzyme; ARBs, angiotensin receptor blockers; PPI, proton pump inhibitor

The lesion characteristics are shown in Table 2. There were 433 lesions that were treated with PCI using drug-eluting stents (88.9%). All stents were third-generation drug-eluting stents.

**Table 2:** Lesion characteristics

Characteristics	Overall (n = 487)
	Number (%) or mean ± SD
Target lesion site	
LMT	13 (2.7)
LAD	242 (49.7)
LCX	91 (18.7)
RCA	139 (28.5)
SVG	2 (0.4)
PCI procedure	
Drug eluting stent	433 (88.9)
Number of stents	1.23 ± 0.50
Average stent diameter (mm)	3.12 ± 0.50
Total stent length (mm)	30.30 ± 17.65
Drug-coated balloon	54 (11.1)

LMT, left main trunk; LAD, left anterior descending; LCX, left circumflex; RCA, right coronary artery; SVG, saphenous vein graft; PCI, percutaneous coronary intervention

### Incidence rates of MACE ① and MACE ②

**Fig. 1:**
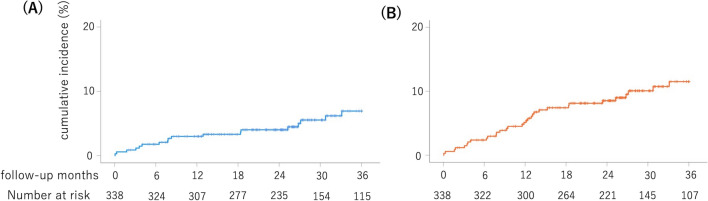
Incidence rates of MACE ① and MACE ②. **A** The cumulative incidence rates for MACE ① (composite of all-cause death, non-fatal myocardial infarction, stent thrombosis, and non-fatal cerebral infarction) at 6, 12, 24, and 36 months were 1.8%, 3.0%, 3.8%, and 5.3%, respectively. **B** The cumulative incidence rates for MACE ② (composite of all-cause death, non-fatal myocardial infarction, stent thrombosis, non-fatal cerebral infarction, and TVR) at 6, 12, 24, and 36 months were 2.4%, 5.0%, 8.0%, and 9.5%, respectively. MACE, major adverse cardiovascular event; TVR, target vessel revascularization

A total of 14 patients died, three developed non-fatal myocardial infarction, one developed cerebral infarction, and 14 underwent TVR during the 36-month follow-up period. Stent thrombosis did not occur. The cumulative incidence rates of MACE ① at 6, 12, 24, and 36 months were 1.8%, 3.0%, 3.8%, and 5.3%, respectively (Fig. 1A). The cumulative incidence rates of MACE ② at 6, 12, 24, and 36 months were 2.4%, 5.0%, 8.0%, and 9.5%, respectively (Fig. 1B).

### Time-dependent ROC curves of the PRU values for MACE ① and MACE ②

**Fig. 2:**
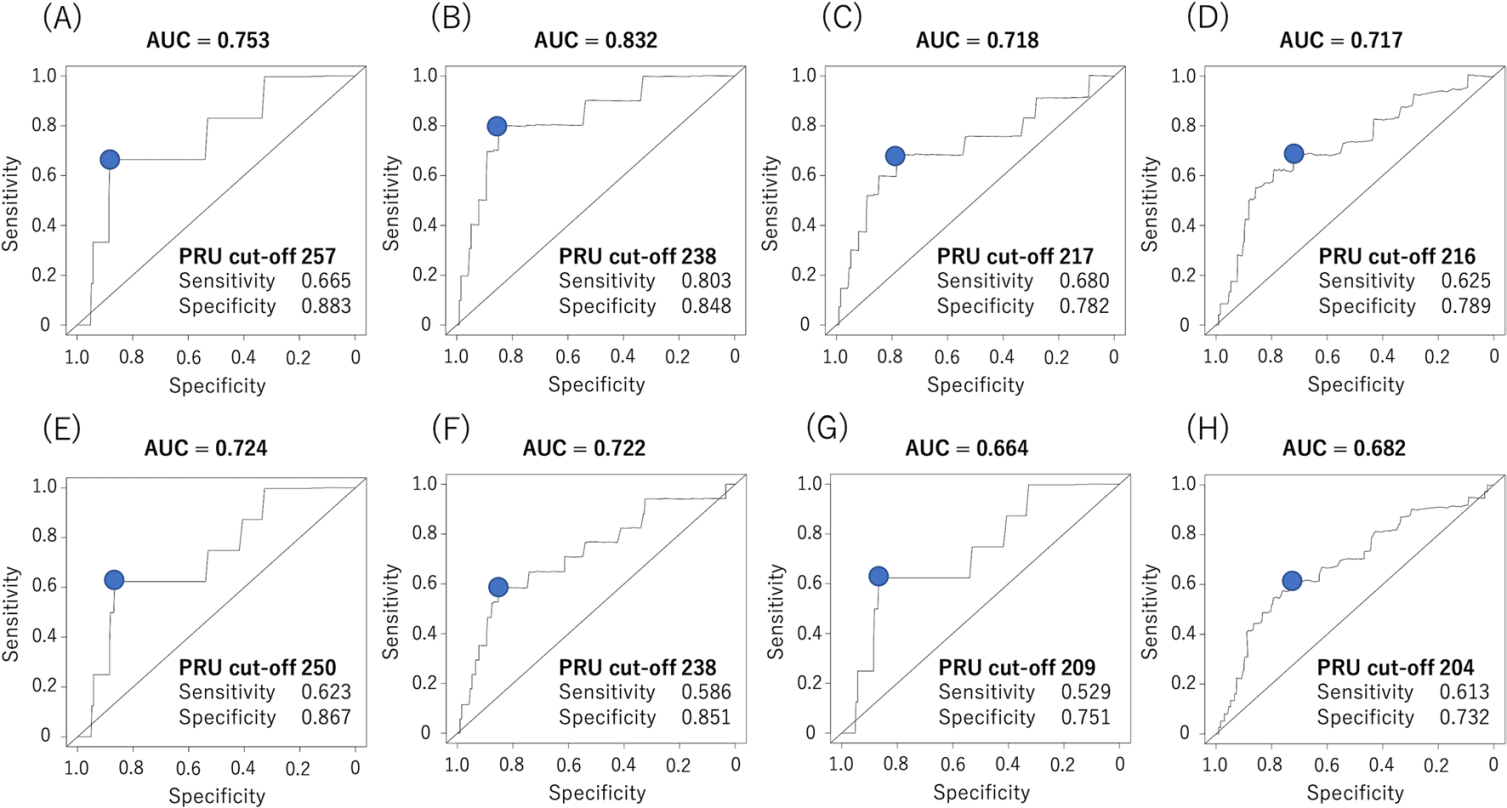
Time-dependent ROC curves of the PRU values for MACE ① and MACE ② at 6, 12, 24, and 36 months during follow-up. **A** Time-dependent ROC curve for MACE ① (composite of all-cause death, non-fatal myocardial infarction, stent thrombosis, and non-fatal cerebral infarction) at 6 months. **B** Time-dependent ROC curve for MACE ① at 12 months. **C** Time-dependent ROC curve for MACE ① at 24 months. **D** Time-dependent ROC curve for MACE ① at 36 months. **E** Time-dependent ROC curve for MACE ② (composite of all-cause death, non-fatal myocardial infarction, stent thrombosis, non-fatal cerebral infarction, and TVR) at 6 months. **F** Time-dependent ROC curve for MACE ② at 12 months. **G** Time-dependent ROC curve for MACE ② at 24 months. **H** Time-dependent ROC curve for MACE ② at 36 months. ROC, receiver operating characteristic; MACE, major adverse cardiovascular event; PRU, P2Y12 reaction unit; TVR, target vessel revascularization

Figure 2 illustrates the ROC curves for MACE ① and MACE ② at 6, 12, 24, and 36 months using time-dependent ROC analysis. The optimal PRU cut-off values for MACE ① at 6, 12, 24, and 36 months were 257, 238, 217, and 216, respectively. The optimal PRU cut-off values for MACE ② at 6, 12, 24, and 36 months were 250, 238, 209, and 204, respectively. The optimal PRU cut-off values differed between MACE ① and MACE ②, except at 12 months. The optimal PRU cut-off values for MACE ① and MACE ② tended to decrease over time. The AUC for MACE ① and MACE ② at 6, 12, 24, and 36 months were 0.753, 0.832, 0.718, and 0.717, and 0.724, 0.722, 0.664, and 0.682, respectively.

### Changes in AUC over time using time-dependent ROC analysis

**Fig. 3:**
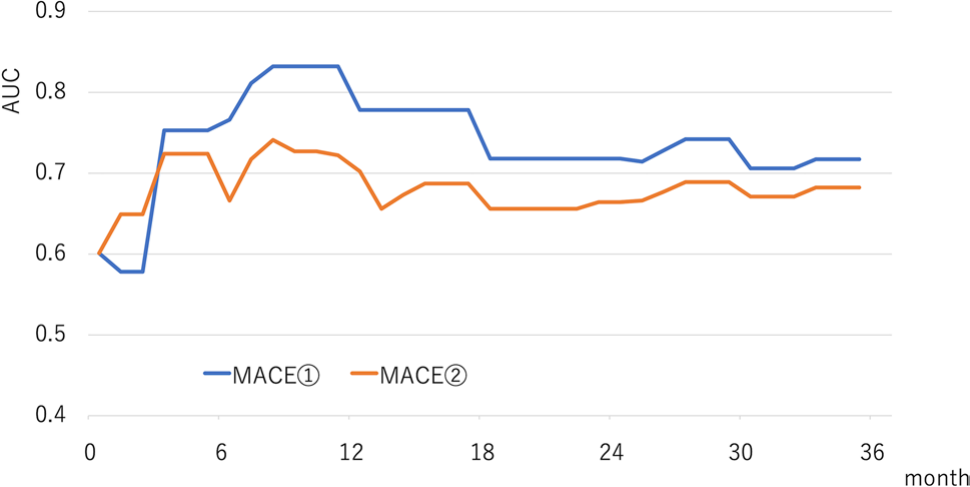
Changes in AUC over time using time-dependent ROC analysis. The AUC of the PRU value for MACE ① (composite of all-cause death, non-fatal myocardial infarction, stent thrombosis, and non-fatal cerebral infarction) and MACE ② (composite of all-cause death, non-fatal myocardial infarction, stent thrombosis, non-fatal cerebral infarction, and TVR) were plotted monthly from the start of follow-up to 36 months. MACE, major adverse cardiovascular event; PRU, P2Y12 reaction unit; TVR, target vessel revascularization; ROC, receiver operating characteristic; AUC, area under the curve

Figure 3 depicts the AUC for MACE ① and MACE ②, plotted monthly by time-dependent ROC analysis. AUC for MACE ② tended be lower than that for MACE ① in most observation periods.

**Fig. 4:**
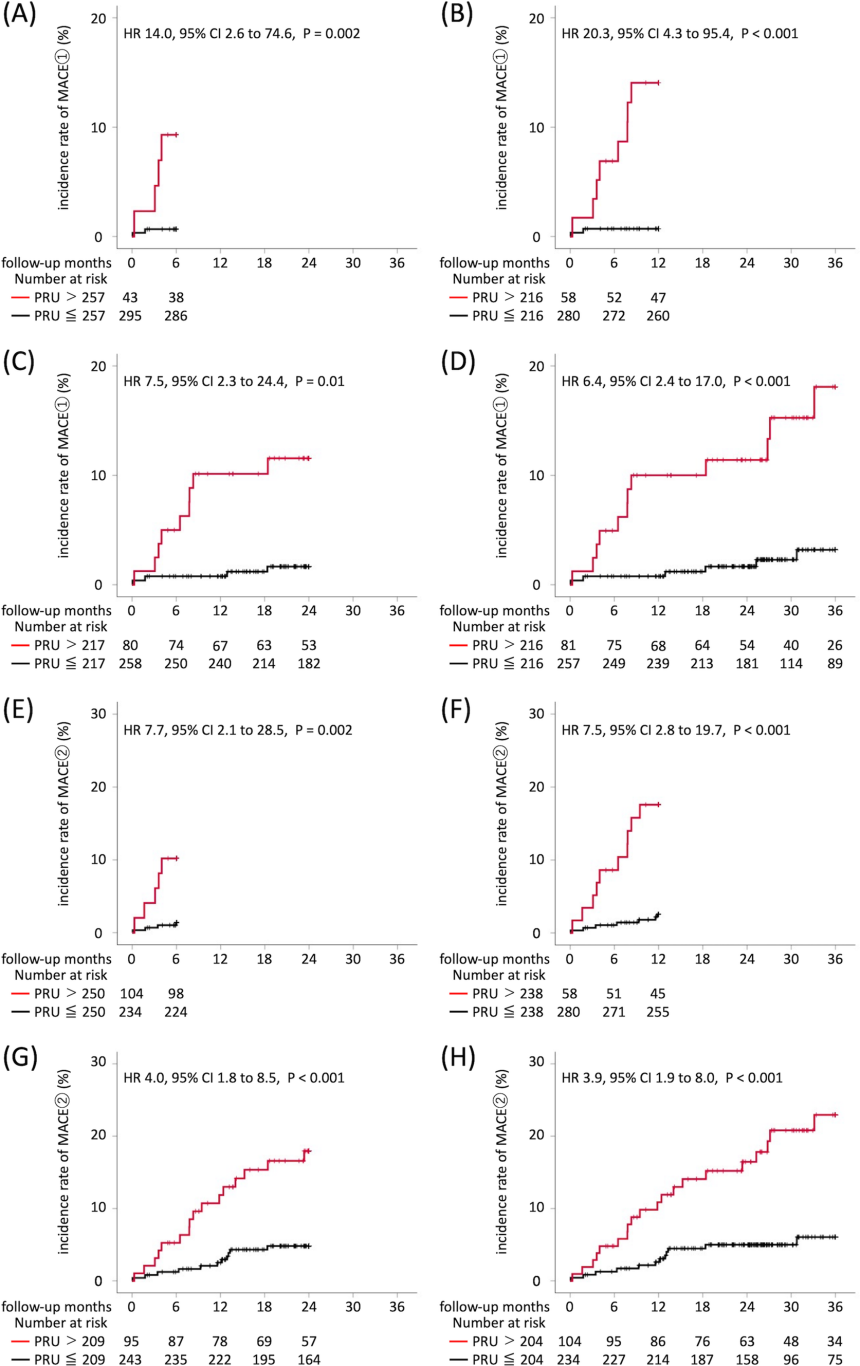
Time-to event curves of MACE ① and MACE ②. By using the time-dependent ROC analysis, the optimal PRU cut-off value for predicting MACE ① (composite of all-cause death, non-fatal myocardial infarction, stent thrombosis, and non-fatal cerebral infarction) and MACE ② (composite of all-cause death, non-fatal myocardial infarction, stent thrombosis, non-fatal cerebral infarction, and TVR) at 6, 12, 24, and 36 months was estimated. Furthermore, the target patients were divided into two groups based on the calculated optimal PRU cut-off value for each period, and the Kaplan–Meier curve was created. **A** Time-to-event curve of MACE ① from the start of follow-up to 6 months. **B** Time-to-event curve of MACE ① from the start of follow-up to 12 months. **C** Time-to-event curve of MACE ① from the start of follow-up to 24 months. **D** Time-to-event curve of MACE ① from the start of follow-up to 36 months. **E** Time-to-event curve of MACE ② from the start of follow-up to 6 months. **F** Time-to-event curve of MACE ② from the start of follow-up to 12 months. **G** Time-to-event curve of MACE ② from the start of follow-up to 24 months. **H** Time-to-event curve of MACE ② from the start of follow-up to 36 months. MACE, major adverse cardiovascular event; HR, hazard ratio; PRU, P2Y12 reaction unit; ROC, receiver operating characteristic; CI, confidence interval; TVR, target vessel revascularization

Figure 4 shows the incidence rates of MACE ① and MACE ② in the two groups divided according to the optimal PRU cut-off values at 6, 12, 24, and 36 months of follow-up. The incidence rate of MACE ① in patients with PRU values above the optimal cut-off value was significantly higher than that in patients at or below the optimal PRU cut-off value in all time periods examined (6 months: 0.7% vs. 9.3%, hazard ratio [HR] 14.0, 95% confidence interval [CI] 2.6–74.6, *P* = 0.002; 12 months: 0.7% vs. 13.8%, HR 20.3, 95% CI 4.3–95.4, *P* < 0.001; 24 months: 1.6% vs. 11.3%, HR 7.5; 95% CI 2.3–24.4, *P* = 0.01; 36 months: 2.3% vs. 14.8%, HR 6.4, 95% CI 2.4–17.0, *P* < 0.001). Similar results were observed in MACE ② (6 months: 1.4% vs. 10.2%, HR 7.7, 95% CI 2.1–28.5, *P* = 0.002; 12 months: 2.5% vs. 17.2%, HR 7.5, 95% CI 2.8–19.7, *P* < 0.001; 24 months: 4.5% vs. 16.8%, HR 4.0, 95% CI 1.8–8.5, *P* < 0.001; 36 months: 5.1% vs. 19.2%; HR 3.9, 95% CI 1.9 –17.0, *P* < 0.001).

### Incidence of major bleeding

Major bleeding events occurred in seven (2.1%) patients during the 36-month follow-up period. There was no statistical difference in bleeding event rates between low and high platelet reactions.

## Discussion

In this study, we evaluated the optimal PRU cut-off value for predicting two endpoints. The optimal PRU cut-off value at 6, 12, 24, and 36 months for MACE ① were 257, 238, 217, and 216, and for MACE ② were 250, 238, 209, and 204, respectively. The optimal PRU cut-off values for both endpoints were high during the early observation period but decreased as time progressed. We confirmed that the optimal PRU cut-off value changed depending on different endpoints and over time. Moreover, the predictive ability of PRU values remained high during the observation period for both endpoints and tended to be higher for MACE ① than for MACE ②. The PRU value is a useful index; however, the predictive ability of the PRU value varies, depending on the endpoint.

Only a few long-term studies over 1 year have investigated the predictive ability of PRU cut-off values for cardiovascular events. In this study, we investigated the optimal PRU cut-off value for cardiovascular events over a long-term observation period of 36 months using time-dependent ROC analysis. The optimal PRU cut-off value at 36 months was 216 for MACE ① and 204 for MACE ②.The long-term optimal PRU cut-off value tended toward 208, which is the commonly used cut-off value for cardiovascular events [8, 11, 13, 15]. Therefore, the results of this study may support those of previous studies. We conducted evaluations over a period of 3 years; however, in the case of longer observation periods like 5 or 10 years, lower PRU values may be necessary to reduce cardiovascular events. Further studies are warranted in the future to clarify this.

The PRU value is an HPR index in patients receiving P2Y12 inhibitors, and several studies have demonstrated an association between HPR, arteriosclerosis, and inflammation. Chirumamilla et al. reported that HPR is associated with greater coronary artery atherosclerotic disease burden and plaque calcification using pre-intervention volumetric intravascular ultrasound imaging. [22]. Gori et al. revealed that HPR was associated with elevated inflammatory cytokine levels in patients with acute coronary syndrome undergoing PCI on dual antiplatelet therapy [23]. Bernlochner et al. reported that HPR was associated with elevated levels of C-reactive protein (CRP), white blood cell count, and fibrinogen in patients with ischemic heart disease undergoing PCI [24]. Hori et al. reported that a high CRP level was associated with mortality in patients with myocardial infarction[25]. The potential reason for PRU values being highly predictive of cardiovascular events and a low PRU value being required for long-term event suppression may be that PRU values are associated with the quality and quantity of atherosclerosis, inflammation, and coagulation [26].

This study revealed that the predictive ability of the PRU value for cardiovascular events varied with different endpoints. The AUC for MACE ②, which comprised MACE ① and TVR, tended to be lower than that for MACE ① at almost all observation periods. TVR was mainly performed at the stable lesion in this study. Stable lesions could have weaker associations with inflammation, thrombosis, and unstable plaque than acute lesion do. Therefore, the addition of TVR to MACE ① may reduce the AUC and impact the predictive ability of the PRU value. Furthermore, TVR might be affected by the PCI or CABG procedures. As a result, we performed a similar analysis using an endpoint with MACE③ (the composite of MACE ① and spontaneous coronary events in TVR). Spontaneous coronary events were defined as those not related to PCI or CABG procedures. MACE③ occurred in 25 patients. The predictive ability of the PRU value for MACE③ was more accurate than that for MACE ② and similar to that for MACE ① (data not shown).

Faster and stronger platelet inhibition is required to prevent the occurrence of thrombotic events, especially during the acute phase of acute coronary syndrome. However, this study showed that long-term event suppression required a lower PRU value than did short-term event suppression. This could be because stent thrombosis did not occur during the follow-up period and there was a low percentage (7.1%) of acute coronary syndrome cases in this study.

In several studies, cardiovascular events were reduced by assessing the results of a platelet function test and appropriately adjusting the dose of P2Y12 inhibitor or switching to other P2Y12 inhibitors in patients with HPR [27–32]. It may be possible to reduce long-term cardiovascular events in patients taking P2Y12 inhibitors, who have a high PRU value, by adjusting the dose of the P2Y12 inhibitor or switching to other P2Y12 inhibitors.

Our study had a few limitations. This study was a single-center retrospective study. Multi-center prospective studies are required to confirm the findings of this study. In addition, the sample size may not be sufficient for this study. We believe that it is important to use a larger sample size in future studies. The choice of P2Y12 inhibitor was left at the discretion of the attending physician; therefore, there may be selection bias in this study. Further, all the patients in this study were Japanese. Reportedly, there are several gene polymorphisms of CYP2C19 in Asians [33]. In particular, CYP2C19 is involved in the metabolism of clopidogrel. Therefore, the PRU cut-off value to predict cardiovascular events may differ among races, thereby reducing the generalizability of these findings. Notably, we used 3.75 mg/day of prasugrel, which is commonly used as the maintenance dose in Japan, but it is smaller than the dose used in other countries.

## Conclusions

This study demonstrated that the optimal cut-off and predictive ability of the PRU value for cardiovascular events varied, depending on different endpoints and duration of the observation period. We showed that a relatively high PRU value is effective for short-term event suppression; however, a low value is required for long-term event suppression.

## Data Availability

Anonymized data can be made available upon reasonable request to the corresponding author.

## References

[1] Bonello L, Tantry US, Marcucci R, Blindt R, Angiolillo DJ, Becker R, Bhatt DL, Cattaneo M, Collet JP, Cuisset T, Gachet C, Montalescot G, Jennings LK, Kereiakes D, Sibbing D, Trenk D, Van Werkum JW, Paganelli F, Price MJ, Waksman R, Gurbel PA, Working Group on High On-Treatment Platelet R (2010). Consensus and future directions on the definition of high on-treatment platelet reactivity to adenosine diphosphate. J Am Coll Cardiol.

[2] Sibbing D, Steinhubl SR, Schulz S, Schomig A, Kastrati A (2010). Platelet aggregation and its association with stent thrombosis and bleeding in clopidogrel-treated patients: initial evidence of a therapeutic window. J Am Coll Cardiol.

[3] Jakubowski JA (2008). The use of the VerifyNow P2Y12 point-of-care device to monitor platelet function across a range of P2Y12 inhibition levels following prasugrel and clopidogrel administration. Thromb Haemost.

[4] Malinin A, Pokov A, Spergling M, Defranco A, Schwartz K, Schwartz D, Mahmud E, Atar D, Serebruany V (2007). Monitoring platelet inhibition after clopidogrel with the VerifyNow-P2Y12(R) rapid analyzer: the VERIfy Thrombosis risk ASsessment (VERITAS) study. Thromb Res.

[5] Varenhorst C, James S, Erlinge D, Braun OO, Brandt JT, Winters KJ, Jakubowski JA, Olofsson S, Wallentin L, Siegbahn A (2009). Assessment of P2Y(12) inhibition with the point-of-care device VerifyNow P2Y12 in patients treated with prasugrel or clopidogrel coadministered with aspirin. Am Heart J.

[6] Brar SS, ten Berg J, Marcucci R, Price MJ, Valgimigli M, Kim HS, Patti G, Breet NJ, DiSciascio G, Cuisset T, Dangas G (2011). Impact of platelet reactivity on clinical outcomes after percutaneous coronary intervention. A collaborative meta-analysis of individual participant data. J Am Coll Cardiol.

[7] Breet NJ, van Werkum JW, Bouman HJ, Kelder JC, Ruven HJ, Bal ET, Deneer VH, Harmsze AM, van der Heyden JA, Rensing BJ, Suttorp MJ, Hackeng CM, ten Berg JM (2010). Comparison of platelet function tests in predicting clinical outcome in patients undergoing coronary stent implantation. JAMA.

[8] Lee SN, Moon D, Sung MK, Moon KW, Yoo KD (2019). Impact of platelet reactivity on long-term prognosis in Korean patients receiving percutaneous coronary intervention. Platelets.

[9] Marcucci R, Gori AM, Paniccia R, Giusti B, Valente S, Giglioli C, Buonamici P, Antoniucci D, Abbate R, Gensini GF (2009). Cardiovascular death and nonfatal myocardial infarction in acute coronary syndrome patients receiving coronary stenting are predicted by residual platelet reactivity to ADP detected by a point-of-care assay: a 12-month follow-up. Circulation.

[10] Matsumaru Y, Kitazono T, Kadota K, Nakao K, Nakagawa Y, Shite J, Yokoi H, Kozuma K, Tanabe K, Akasaka T, Shinke T, Ueno T, Hirayama A, Uemura S, Kuroda T, Takita A, Harada A, Iijima R, Murakami Y, Saito S, Nakamura M (2022). Relationship between platelet aggregation and stroke risk after percutaneous coronary intervention: a PENDULUM analysis. Heart Vessels.

[11] Nakamura M, Kadota K, Takahashi A, Kanda J, Anzai H, Ishii Y, Shibata Y, Yasaka Y, Takamisawa I, Yamaguchi J, Takeda Y, Harada A, Motohashi T, Iijima R, Uemura S, Murakami Y, Investigators* PR (2020). Relationship between platelet reactivity and ischemic and bleeding events after percutaneous coronary intervention in East Asian patients: 1-year results of the PENDULUM registry. J Am Heart Assoc.

[12] Patti G, Nusca A, Mangiacapra F, Gatto L, D'Ambrosio A, Di Sciascio G (2008). Point-of-care measurement of clopidogrel responsiveness predicts clinical outcome in patients undergoing percutaneous coronary intervention results of the ARMYDA-PRO (Antiplatelet therapy for Reduction of MYocardial Damage during Angioplasty-Platelet Reactivity Predicts Outcome) study. J Am Coll Cardiol.

[13] Price MJ, Angiolillo DJ, Teirstein PS, Lillie E, Manoukian SV, Berger PB, Tanguay JF, Cannon CP, Topol EJ (2011). Platelet reactivity and cardiovascular outcomes after percutaneous coronary intervention: a time-dependent analysis of the Gauging Responsiveness with a VerifyNow P2Y12 assay: Impact on Thrombosis and Safety (GRAVITAS) trial. Circulation.

[14] Buch AN, Singh S, Roy P, Javaid A, Smith KA, George CE, Pichard AD, Satler LF, Kent KM, Suddath WO, Waksman R (2007). Measuring aspirin resistance, clopidogrel responsiveness, and postprocedural markers of myonecrosis in patients undergoing percutaneous coronary intervention. Am J Cardiol.

[15] Stone GW, Witzenbichler B, Weisz G, Rinaldi MJ, Neumann FJ, Metzger DC, Henry TD, Cox DA, Duffy PL, Mazzaferri E, Gurbel PA, Xu K, Parise H, Kirtane AJ, Brodie BR, Mehran R, Stuckey TD (2013). Platelet reactivity and clinical outcomes after coronary artery implantation of drug-eluting stents (ADAPT-DES): a prospective multicentre registry study. Lancet.

[16] Legrand V, Cuisset T, Chenu P, Vrolix M, Martinez C, Dens J, Gach O, Boland J, Claeys MJ, Magne J, Barbato E, Wijns W (2014). Platelet reactivity and cardiovascular events after percutaneous coronary intervention in patients with stable coronary artery disease: the Stent Thrombosis In Belgium (STIB) trial. EuroIntervention.

[17] Yu LH, Kim MH, Zhang HZ, Park JS, Park TH, Kim YD, Cha KS, Han JY (2012). Impact of platelet function test on platelet responsiveness and clinical outcome after coronary stent implantation: platelet responsiveness and clinical outcome. Korean Circ J.

[18] Heagerty PJ, Lumley T, Pepe MS (2000). Time-dependent ROC curves for censored survival data and a diagnostic marker. Biometrics.

[19] Thygesen K, Alpert JS, Jaffe AS, Chaitman BR, Bax JJ, Morrow DA, White HD, Thygesen K, Alpert JS, Jaffe AS, Chaitman BR, Bax JJ, Morrow DA, White HD, Mickley H, Crea F, Van de Werf F, Bucciarelli-Ducci C, Katus HA, Pinto FJ, Antman EM, Hamm CW, De Caterina R, Januzzi JL, Apple FS, Alonso Garcia MA, Underwood SR, Canty JM, Lyon AR, Devereaux PJ, Zamorano JL, Lindahl B, Weintraub WS, Newby LK, Virmani R, Vranckx P, Cutlip D, Gibbons RJ, Smith SC, Atar D, Luepker RV, Robertson RM, Bonow RO, Steg PG, O’Gara PT, Fox KAA, Hasdai D, Aboyans V, Achenbach S, Agewall S, Alexander T, Avezum A, Barbato E, Bassand J-P, Bates E, Bittl JA, Breithardt G, Bueno H, Bugiardini R, Cohen MG, Dangas G, de Lemos JA, Delgado V, Filippatos G, Fry E, Granger CB, Halvorsen S, Hlatky MA, Ibanez B, James S, Kastrati A, Leclercq C, Mahaffey KW, Mehta L, Müller C, Patrono C, Piepoli MF, Piñeiro D, Roffi M, Rubboli A, Sharma S, Simpson IA, Tendera M, Valgimigli M, van der Wal AC, Windecker S, Chettibi M, Hayrapetyan H, Roithinger FX, Aliyev F, Sujayeva V, Claeys MJ, Smajić E, Kala P, Iversen KK, El Hefny E, Marandi T, Porela P, Antov S, Gilard M, Blankenberg S, Davlouros P, Gudnason T, Alcalai R, Colivicchi F, Elezi S, Baitova G, Zakke I, Gustiene O, Beissel J, Dingli P, Grosu A, Damman P, Juliebø V, Legutko J, Morais J, Tatu-Chitoiu G, Yakovlev A, Zavatta M, Nedeljkovic M, Radsel P, Sionis A, Jemberg T, Müller C, Abid L, Abaci A, Parkhomenko A, Corbett S (2019). Fourth universal definition of myocardial infarction (2018). Eur Heart J.

[20] Mehran R, Rao SV, Bhatt DL, Gibson CM, Caixeta A, Eikelboom J, Kaul S, Wiviott SD, Menon V, Nikolsky E, Serebruany V, Valgimigli M, Vranckx P, Taggart D, Sabik JF, Cutlip DE, Krucoff MW, Ohman EM, Steg PG, White H (2011). Standardized bleeding definitions for cardiovascular clinical trials: a consensus report from the Bleeding Academic Research Consortium. Circulation.

[21] Kanda Y (2013). Investigation of the freely available easy-to-use software 'EZR' for medical statistics. Bone Marrow Transpl.

[22] Chirumamilla AP, Maehara A, Mintz GS, Mehran R, Kanwal S, Weisz G, Hassanin A, Hakim D, Guo N, Baber U, Pyo R, Moses JW, Fahy M, Kovacic JC, Dangas GD (2012). High platelet reactivity on clopidogrel therapy correlates with increased coronary atherosclerosis and calcification: a volumetric intravascular ultrasound study. JACC Cardiovasc Imaging.

[23] Gori AM, Cesari F, Marcucci R, Giusti B, Paniccia R, Antonucci E, Gensini GF, Abbate R (2009). The balance between pro- and anti-inflammatory cytokines is associated with platelet aggregability in acute coronary syndrome patients. Atherosclerosis.

[24] Bernlochner I, Steinhubl S, Braun S, Morath T, Jaitner J, Stegherr J, Mehilli J, von Beckerath N, Schömig A, Kastrati A, Sibbing D (2010). Association between inflammatory biomarkers and platelet aggregation in patients under chronic clopidogrel treatment. Thromb Haemost.

[25] Hori Y, Sakakura K, Jinnouchi H, Taniguchi Y, Tsukui T, Watanabe Y, Yamamoto K, Seguchi M, Wada H, Fujita H (2023). Association of peak C-reactive protein with long-term clinical outcomes in patients with ST-segment elevation myocardial infarction. Heart Vessels.

[26] Tantry US, Bonello L, Aradi D, Price MJ, Jeong YH, Angiolillo DJ, Stone GW, Curzen N, Geisler T, Ten Berg J, Kirtane A, Siller-Matula J, Mahla E, Becker RC, Bhatt DL, Waksman R, Rao SV, Alexopoulos D, Marcucci R, Reny JL, Trenk D, Sibbing D, Gurbel PA, Working Group on On-Treatment Platelet R (2013). Consensus and update on the definition of on-treatment platelet reactivity to adenosine diphosphate associated with ischemia and bleeding. J Am Coll Cardiol.

[27] Bagai A, Peterson ED, McCoy LA, Effron MB, Zettler ME, Stone GW, Henry TD, Cohen DJ, Schulte PJ, Anstrom KJ, Wang TY (2017). Association of measured platelet reactivity with changes in P2Y(12) receptor inhibitor therapy and outcomes after myocardial infarction: Insights into routine clinical practice from the TReatment with ADP receptor iNhibitorS: Longitudinal Assessment of Treatment Patterns and Events after Acute Coronary Syndrome (TRANSLATE-ACS) study. Am Heart J.

[28] Bonello L, Camoin-Jau L, Armero S, Com O, Arques S, Burignat-Bonello C, Giacomoni MP, Bonello R, Collet F, Rossi P, Barragan P, Dignat-George F, Paganelli F (2009). Tailored clopidogrel loading dose according to platelet reactivity monitoring to prevent acute and subacute stent thrombosis. Am J Cardiol.

[29] Bonello L, Camoin-Jau L, Arques S, Boyer C, Panagides D, Wittenberg O, Simeoni MC, Barragan P, Dignat-George F, Paganelli F (2008). Adjusted clopidogrel loading doses according to vasodilator-stimulated phosphoprotein phosphorylation index decrease rate of major adverse cardiovascular events in patients with clopidogrel resistance: a multicenter randomized prospective study. J Am Coll Cardiol.

[30] Christ G, Siller-Matula JM, Francesconi M, Dechant C, Grohs K, Podczeck-Schweighofer A (2014). Individualising dual antiplatelet therapy after percutaneous coronary intervention: the IDEAL-PCI registry. BMJ Open.

[31] Hazarbasanov D, Velchev V, Finkov B, Postadjian A, Kostov E, Rifai N, Aradi D (2012). Tailoring clopidogrel dose according to multiple electrode aggregometry decreases the rate of ischemic complications after percutaneous coronary intervention. J Thromb Thrombolysis.

[32] Siller-Matula JM, Francesconi M, Dechant C, Jilma B, Maurer G, Delle-Karth G, Gouya G, Ruzicka K, Podczeck-Schweighofer A, Christ G (2013). Personalized antiplatelet treatment after percutaneous coronary intervention: the MADONNA study. Int J Cardiol.

[33] Jang JS, Cho KI, Jin HY, Seo JS, Yang TH, Kim DK, Kim DS, Seol SH, Kim DI, Kim BH, Park YH, Je HG, Jeong YH, Lee SW (2012). Meta-analysis of cytochrome P450 2C19 polymorphism and risk of adverse clinical outcomes among coronary artery disease patients of different ethnic groups treated with clopidogrel. Am J Cardiol.

